# Conflict in a word‐based approach‐avoidance task is stronger with positive words

**DOI:** 10.1002/brb3.3008

**Published:** 2023-05-11

**Authors:** Johannes Klackl, Jens Blechert, Eva Jonas

**Affiliations:** ^1^ Department of Psychology Paris‐Lodron University of Salzburg Hellbrunnerstrasse Salzburg Austria; ^2^ Centre for Cognitive Neuroscience Paris‐Lodron University of Salzburg

**Keywords:** approach, avoidance, event‐related potential, manikin task, motivation

## Abstract

**Background:**

Valence and motivational direction are linked. We approach good things and avoid bad things, and experience overriding these links as conflicting. Positive valence is more consistently linked with approach than negative valence is linked with avoidance. Therefore, avoiding positive stimuli should produce greater behavioral and neural signs of conflict than approaching negative stimuli.

**Methods:**

In the present event‐related potential study, we tested this assumption by contrasting positive and negative conflict. We used the manikin task, in which we read positive and negative words that they needed to approach and avoid.

**Results:**

Consistent with our prediction, positive conflict prolonged reaction times more than negative conflict did. A late (500–1000 ms following word onset) event‐related potential that we identified as the Conflict slow potential, was only sensitive to positive conflict.

**Conclusion:**

The results of this study support the notion that avoiding positive stimuli is more conflicting than approaching negative stimuli. The fact that the conflict slow potential is typically sensitive to response conflict rather than stimulus conflict suggests that the manikin task primarily requires people to override prepotent responses rather than to identify conflicting stimuli. Thus, the present findings also shed light on the psychological processes subserving conflict resolution in the manikin task.

## Graphical abstract

The manikin task requires overriding intuitive responses to approach and avoid valent stimuli. Avoiding vs. aproaching positive stimuli boosted the N450 and the conflict slow potential. We observed no conflict effect with negative words, presumably because negative valence is less associated with avoidance than positive valence is associated with approach.

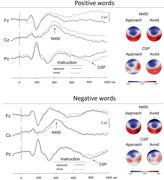

## 1 INTRODUCTION

Human information processing and behavior are remarkably automatic in nature. We are faster to approach pleasant and avoid unpleasant things than to approach unpleasant and avoid pleasant things (Chen & Bargh, 1999; Krieglmeyer & Deutsch, 2010; Solarz, 1960). This phenomenon has been proposed to result from two cognitive processes: Incoming stimuli are classified as either positive or negative; in turn, these stimuli elicit corresponding approach and avoidance behavioral tendencies (Bargh & Chartrand, 1999; Chen & Bargh, 1999; Lang & Bradley, 2015; Neumann et al., 2003). Together, these two processes allow us to make decisions quickly and with little mental effort. People are drawn to what is pleasant and repelled from what is unpleasant.

In order not to lose sight of long‐term plans and desires, we often need to override these automatic, or “default” tendencies (Baumeister & Heatherton, 1996). For example, to maintain or reduce body weight, we need to avoid eating certain pleasant foods, including junk food and desserts; to maintain dental health, we need to perform the boring daily ritual of brushing and flossing our teeth, and sometimes even endure unpleasant treatments while seated in a dentist's chair. Ingrained in these situations is a conflict between wanting to approach or avoid something and “shoulding” the opposite (Carver, 2005; Hofmann et al., 2009). Being able to override automatic, impulsive tendencies is an important component of self‐control (Baumeister & Heatherton, 1996) which ultimately improves physical and mental health, well‐being, and life satisfaction (Hofmann et al., 2014).

These automatic, or “default” tendencies to approach what is positive and avoid what is negative, as well as the effort that it takes to override them, have been extensively studied using approach‐avoidance tasks. These tasks typically feature two types of trials. In congruent trials, participants need to act intuitively (i.e., approaching positive and avoiding negative stimuli). The incongruent trials require counterintuitive actions (approaching negative and avoiding positive stimuli). People usually respond faster on congruent trials, which is presumably because overriding the intuitive choices requires effort (for a review, see Phaf et al., 2014). This effect is often referred to as the stimulus‐response compatibility (SRC) effect. SRC effects are well documented in many contexts, including positive versus negative words (Chen & Bargh, 1999; Krieglmeyer & Deutsch, 2010; Solarz, 1960), spider pictures versus spider‐free pictures among spider phobics (Rinck & Becker, 2007), pictures of people with AIDS versus pictures of healthy people (Neumann et al., 2004), pictures of appetitive foods versus objects (van Alebeek et al., 2022), and smoking‐related versus smoking‐unrelated pictures among smokers (Mogg et al., 2003).

In approach‐avoidance tasks with positive and negative stimuli, conflict can stem from avoiding positive stimuli (hereafter called “positive conflict”) and approaching negative stimuli (hereafter called “negative conflict”). We argue that SRCs with positive stimuli should be larger than SRCs with negative stimuli because positive emotions are more strongly linked to approach than negative emotions are linked to avoidance. Approaching is the default response tendency to positive stimuli, but there is no clear default tendency for negative stimuli (Carver, 2005). While positive stimuli call for approach (e.g., reaching out toward, walking toward, and searching), negative stimuli may call for either passive avoidance (e.g., waiting, freezing, inhibition) or active avoidance (e.g., evading, dodging, rejecting, refusing, fleeing) (Fanselow, 1994; Veling et al., 2008). Sometimes, negative stimuli even call for approach, as the case of anger shows. Anger is a negatively valent yet approach‐related emotion (Carver & Harmon‐Jones, 2009; Harmon‐Jones, 2004, 2010). Positive and negative conflicts also differ with regard to the psychological phenomena that emerge when people actually succeed at overriding their intuitive tendencies. Deciding to approach undesirable things often requires overcoming anxiety, fear, disgust, or anger. Deciding to avoid something pleasant is associated with frustrative nonreward and requires dealing with disappointment and sadness about having lost pleasure. Taken together, avoiding positive stimuli is different from approaching negative stimuli.

Psycholinguistic studies have confirmed the notion that positive and negative valence differ by showing that negative affective language is more diversified, whereas positive affective language is more alike. Words that express negative emotions outnumber those that express pleasant emotions, and this is because of the need to clearly characterize and classify negative states as opposed to positive states (Semin & Fiedler, 1991; Schrauf & Sanchez, 2004). When presented in pairs, positive words are perceived as more similar than negative words (Unkelbach et al., 2008). Together, positive information is more similar and more densely organized in language and memory than negative information. These basic differences between how positive and negative valence are represented in our minds add another reason to believe that approaching something negative is not the same as avoiding something positive.

Although many studies have used approach‐avoidance tasks with positive and negative stimuli, few have explicitly hypothesized that SRCs are greater with positive than with negative stimuli. Nevertheless, there is evidence for this hypothesis. Stins et al. (2011) found that participants were faster at stepping forward toward a happy face than stepping back from a happy face; however, they stepping back from an angry face took roughly as long as stepping toward it. Ascheid et al. (2019) found that participants approached positive pictures faster than they avoided them, but did not avoid negative pictures faster than they approached them. In a recent study (van Alebeek et al., 2022), people approached positive pictures of appetitive foods and butterflies faster than they avoided them, but they did not avoid negative stimuli (pictures of spoiled foods and spiders) faster than they approached them. Ratings of food palatability correlate with the speed of aproach trials but not with the speed of avoidance trials in two studies (Kahveci et al., 2021; van Alebeek et al., 2022).

### 1.1 Neurophysiological aspects

Neuroscientific evidence suggests that our capacity to override impulsive tendencies critically depends on a tandem system of the anterior cingulate and left prefrontal cortex (Botvinick et al., 2001, 2004; Kerns et al., 2004; MacDonald et al., 2000; Miller & Cohen, 2001; O'Reilly et al., 2010; Perlstein et al., 2006). The anterior cingulate cortex detects conflicts, and thereby signals an increased need to regulate intuitive or default impulses (Van Veen & Carter, 2002; Yeung et al., 2004). For example, the ACC becomes active when a chocolate lover who is also trying to eat less chocolate for health reasons, is being offered chocolate, and perceives a conflict between the desire to eat chocolate, and their goal of avoiding it. The lateral prefrontal cortex brings behavior in line with goals, which is called regulation (or resolution). Regulation includes implementing top‐down control, (re)allocating attentional resources, and overriding task‐inappropriate response tendencies. Regulative control is likely involved when we try to override automatic and intuitive tendencies to approach or avoid something. The chocolate lover's success in sticking to their health goals should (at least partially) be determined by how effectively their prefrontal‐based regulation works.

Much neuroscientific evidence on how the brain handles conflict comes from paradigms that do not differentiate between avoiding and approaching. Examples are the Go/No‐go, Stop‐signal, Flanker, and the Stroop task (Gratton et al., 2018). The Go/No‐go task requires participants to respond to some stimuli while withholding their response to others. The Stop‐signal task requires participants to respond quickly unless a stop signal arrives. In the Eriksen flanker task, an arrow is surrounded by other arrows that either point in the same direction (congruent flankers) or in the opposite direction (incongruent flankers). The Stroop task requires participants to name the color of color words (e.g., red, green) that are written in that color (consistent trials) or a different color (inconsistent trials).

Although all of these tasks are similar in that they manipulate the presence of conflict, they differ with regard to whether that conflict arises at the stimulus or at the response level. It is reasonable to assume that in conflict tasks, people first process the stimuli that they encounter, and then generate a behavioral response. Conflict at the stimulus level may arise because some dimensions of the stimuli differ. On an incongruent Stroop trials, a word (e.g., “green”) and the color in which it is written are in conflict. Conflict at the response level may arise because different motor responses compete to determine overt behavior. For example, the word “Green” written in red may produce vocalizations of the words “Green” and “Red” that compete for execution (Hock & Egeth, 1970; Szücz & Soltéz, 2012).

In approach‐avoidance tasks like the one used in this study, conflict is likely to arise solely at the response level. In the incongruent conditions of the task (approaching negative and avoiding positive), the task instructions dictate that participants do not follow the automatic tendency to approach positive and avoid negative, but to do the opposite. Because positive valence is linked to approach, participants need to work hard to override that default tendency. Because the link between negative affect and avoidance is weaker to begin with, participants need to work less hard to override it. Conflict at the stimulus level is unlikely to arise in the approach‐avoidance task used here because the stimuli themselves are not inherently conflicting.^1^

ERP studies typically find that in tasks, response‐level conflict produces a conflict slow potential (CSP), whereas conflict at the stimulus level tends to produce an N450 (Heidlmayr et al., 2020; Larson et al., 2014; Szücz & Soltéz, 2012). Based on these findings, we expect conflict in the manikin task to evoke these components rather than stimulus conflict‐sensitive components.

### 1.2 Positive conflict > negative conflict?

Existing studies that relied on approach‐avoidance tasks have already found that conflict is greater with positive stimuli. Ernst et al. (2013) reported that avoiding rather than approaching positive pictures led to enhanced N1 and N2 amplitudes, but no such effect was apparent with negative images. In another experiment (Bamford et al., 2015), approaching rather pleasant images was associated with a larger late positive potential (350–930 ms) than avoiding pleasant images. Although a similar effect was apparent with negative stimuli, that effect tended to be smaller and nonsignificant. An exception to this rule is a study by van Peer et al. (2007) that found that angry faces elicited smaller N2 (yet larger P150 and P3) amplitudes when participants had to approach them rather than avoid them. However, this effect was only observed in anxious participants to whom cortisol was administered, indicating that this effect may not be encountered under normal circumstances.

### 1.3 The present study

Our hypothesis is that stimulus‐congruency effects (SRCs) are stronger and more reliable with positive stimuli than with negative stimuli because positive valence is more consistently linked to approach than negative valence is linked to avoidance. We tested this hypothesis using the manikin task, which requires participants to move a manikin on the screen towards and away from positive and negative stimuli (De Houwer et al., 2001; Enaida et al., 2013). It differs from most other approach‐avoidance tasks in that it does not require the use of joysticks, levers, or other devices to exert pushing or arm extension movements or pulling or arm flexion movements to indicate avoidance and approach (Phaf et al., 2014). The manikin task requires participants to move a manikin on the screen toward and away from positive and negative stimuli by pressing buttons on a regular computer keyboard (De Houwer et al., 2001; Mogg et al., 2003). This eliminates the need for specialized equipment such as joysticks or levers. It also reduces the risk that participants adapt to the incompatible task requirements by imagining reaching out to touch the positive stimuli via “pushing” movements, and withdrawing their hands from negative stimuli using “pulling” movements (Krieglmeyer & Deutsch, 2010)⁠. Such adaptations may help participants make faster responses yet artificially reduce SRC effects. For these and/or other reasons, the manikin task is more sensitive to automatic approach and avoidance tendencies than tasks requiring pushing and pulling movements (De Houwer et al., 2001; Krieglmeyer & Deutsch, 2010)⁠.

Because the stimuli in this task are not conflicting per se, conflict is likely to arise at the response level. In other words, participants need to inhibit the automatic tendency of approaching positive and avoiding negative stimuli. Since the former is harder than the latter, we expect positive conflict to enhance the CSP, an event‐related potential (ERP) that has been consistently linked to response conflict.

## 2 MATERIALS AND METHODS

### 2.1 Participants

Ninety‐two participants (63 females, 20 males, nine undisclosed), aged 23.04 years (*SD* = 4.05) participated in the experiment. The required sample size was calculated based on a small effect size (*d* = 0.30), an alpha error probability of .05 and a power of .80. Participants were rewarded with money or partial course credit. The study protocol was approved by the ethical review board of the Paris‐Lodron University of Salzburg.

### 2.2 Task and design

**FIGURE 1:**
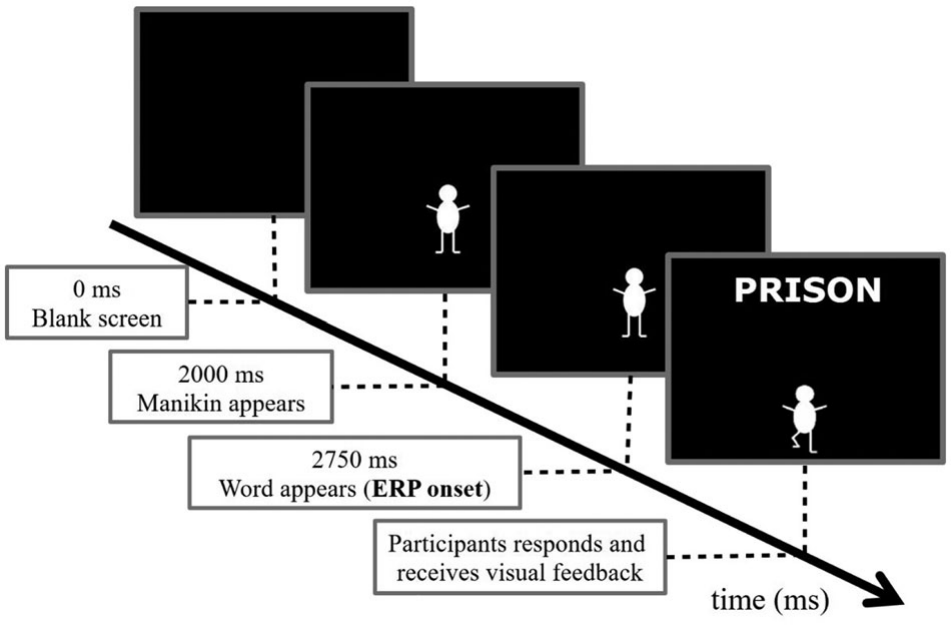
An illustration of a typical manikin task trial.

We used the Manikin task to manipulate stimulus valence (positive, negative; within participants) and movement direction (approach, avoid; within participants). On each trial, a manikin appeared above or below the center of the screen (see Figure 1). After 750 ms, a positive or negative target word appeared at the opposite location. Reaction times and ERPs were referenced to the onset of the target word. When the manikin appeared below the center, the target word always appeared above the center, and vice versa. Participants moved the manikin toward or away from the target word by pressing the up or down button on a numpad. Thus, the task can be regarded a ‘feature relevant’ task, in that attention was directed to stimulus valence (Phaf et al., 2014). Positive and negative words appeared equally often above and below the center of the screen. The intertrial interval lasted 2 s. The 120 trials were divided into two blocks of equal size. In the compatible block, participants had to move the manikin towards positive words and away from negative words. In the incompatible block, participants were instructed to move the manikin away from positive and toward negative words. The order of the incompatible and compatible blocks was randomized across participants. We instructed participants to respond as fast as possible without making any mistakes. We used Inquisit 4.0.8 (Millisecond, 2014) on a 21‐inch screen with a 1980 × 1080 pixel resolution. Participants were familiarized themselves with the task using 10 trials during which they received visual feedback about whether their responses were correct. In the actual trials of the task, no feedback was given.

### 2.3 Stimuli

Following Krieglmeyer and Deutsch (2010), we used 30 positive and 30 negative German words (Hager & Hasselhorn, 1994; Klauer & Musch, 1999). All words are listed in Supporting Information Table S1. We conducted an online pretest (*n* = 30) to see whether the positive and negative words were suitable. Participants rated each word with a valence scale ranging from −3 (very negative) to 3 (very positive). The positive words were indeed rated as positive (*M* = 1.97, *SD* = 1.10), and the negative words were rated as negative (*M* = −2.18, *SD* = 0.99). The difference between these means was significant, *t*(58) = 38.08, *p* < .001. Both positive and negative words were moderately high on a 5‐point arousal scale ranging from 1 (not arousing at all) to 5 (extremely arousing): Positive words: *M* = 2.93, *SD* = 1.30; negative words: *M* = 2.59, *SD* = 1.43). The difference between these means was also significant, *t*(58) = 3.057, *p* = .003. In summary, the positive words were rated as clearly positive, and the negative words were rated as clearly negative. In addition, the positive words were rated as more arousing, but the latter difference was small (0.34 on a 5‐point scale).

### 2.4 Procedure

The manikin task was part of a larger electroencephalograms (EEG) study. Participants performed the manikin task as the third of four unrelated tasks. The other tasks were a mortality salience manipulation, a hand contraction task, and a visual n‐back task. Following the tasks, participants filled out several personality questionnaires: personal project zeal, ethnocentrism, promotion‐prevention regulatory focus strength, mindfulness, aggression, neuroticism, the need for cognitive closure, anxiety and approach motivation (unpublished scales developed in‐house), anxiety, self‐efficacy, and self‐esteem. Last, handedness and demographic data were assessed, and participants were debriefed.

### 2.5 EEG recording and analysis

Throughout the task, we recorded participants’ EEG using a REFA 8–72 digital amplifier system (TMSi, Oldenzaal, The Netherlands). EEG signals were referenced against the average of all scalp electrodes. A wet band on the left wrist served as a ground electrode. An EEG cap with 64 Ag/AgCl‐electrodes were placed on the following locations (according to the international 10–20 system): AF3, AF4, AF7, AF8, AFz, C1, C2, C3, C4, C5, C6, CP1, CP2, CP3, CP4, CP5, CP6, CPz, Cz, F1, F2, F3, F4, F5, F6, F7, F8, FC1, FC2, FC3, FC4, FC5, FC6, FCz, Fp1, Fp2, FT7, FT8, FT9, FT10, Fz, O1, O2, Oz, P1, P2, P3, P4, P5, P6, P7, P8, PO3, PO4, PO7, PO8, POz, Pz, T7, T8, TP7, TP8, TP9, and TP10. We recorded vertical EOG using a bipolar electrode pair placed above and below the left eye. Electrode impedances were kept below 50 kΩ, which is appropriate for this kind of high‐input impedance amplifier (Ferree et al., 2001). Data collection was controlled by Polybench software 1.25 (TMSi). EEG data were digitized at 512 Hz sample rate. Offline analysis of the EEG data was conducted using BrainVisionAnalyzer (BrainProducts, Inc., NC, USA). We applied a 0.1 Hz high‐pass, a 40 Hz low‐pass, and a 50 Hz notch filter. We created segments ranging from 100 ms before until 1 s after the onset of each target word. We used independent component analysis to manually identify and remove eyeblink‐ and eye movement‐related activity. We manually identified the relevant components. An automatic artifact rejection excluded trials with voltage steps greater than 5 μV/ms, more than 70 μV difference between two measurement points within 300 ms, and less than 0.5 μV signal change within 100 ms. For the single trials, epochs ranging from −100 to 1000 ms relative to the stimulus were created, rendering a total of 60 segments for each participant. Finally, single trial ERPs were averaged across the four task conditions: approach positive, avoid positive, approach negative, avoid negative.

On average, positive approach averages consisted of 10.65 single‐trial ERPs (SD = 3.62). Positive avoidance averages consisted of 10.52 single‐trial ERPs (SD = 3.98). Negative avoidance averages consisted of 10.06 single‐trial ERPs (*SD* = 3.88). Negative approach averages consisted of 11.30 single‐trial ERPs (SD = 3.83). Although these numbers are relatively low, they should be high enough to detect the effect of interest with a satisfactory amount of power. According to Monte Carlo simulations by Boudewyn et al. (2018), a within‐participants condition difference of 2 microvolts (which roughly corresponds to the condition difference obtained in our study) can be detected with close to 100% power with 16 trials per condition and 32 participants. At eight trials per condition, that figure is 40%. Considering that our sample was considerably larger, and that we had about 11 trials per condition, we estimate that our achieved statistical power ranges somewhere between these two figures, and should be comparable to the long‐standing convention of 80% (Cohen, 1992). The data that support the findings of this study are available from the corresponding author upon reasonable request.

#### 2.5.1 Stimulus‐evoked waveforms

Visual inspection of the stimulus‐locked grand average ERPs revealed four main components. A visual P1 emerged at occipital and parieto‐occipital electrodes, peaking roughly at 125 ms after stimulus onset. At 200 ms, a parieto‐occipital N1 emerged. A N450 emerged at central sites. From 500 to 1000 ms, we observed a CSP at centroparietal scalp locations. Because the CSP is usually in the positive voltage range yet negative going (Larson et al., 2014), smaller values indicate more neural activity. Like in previous ERP studies using the Stroop task (Hanslmayr et al., 2008; Liotti et al., 2000; West, 2003), the CSP coincided with a simultaneous frontal, positive‐going negativity that mirrored the incongruency effects. For parsimony and because the effects of the frontal deflection mirrored those of the centroparietal deflection, we reported only the posterior deflection. For the statistical analyses, we extracted the mean voltage at the following electrodes and time windows: P1: 100–150 ms at electrodes O1, Oz, O2, PO7, and PO8; N1: 160–220 ms at electrodes P1, Pz, and P2; N450: 250–500 ms at C1, Cz, and C2; conflict SP: 500–1000 ms at electrodes CP1, CPz, and CP2).

#### 2.5.2 Data analysis

Participants’ hit rates, reaction times, and amplitude strengths in the four conditions of the task were subjected to ANOVAs with valence and direction as within‐subject factors. There was a partial loss in the behavioral data of one participant. EEG data of 10 participants were lost due to technical problems including malfunctions in marker signal recording and poor data quality. Partial data loss occurred in six additional participants (1, 2, or 3 conditions out of 4). We used paired sample *t*‐tests to quantify the effects of positive conflict (i.e., avoid positive vs. approach positive) and negative conflict (i.e., approach negative vs. avoid negative). We corrected the *p*‐values for multiple comparisons using Sidak's (1967) procedure.

## 3 RESULTS

### 3.1 Behavioral results

#### 3.1.1 Accuracy

Participants responded most accurately with hit rates of more than 90% (see Table 1). The linear mixed model of participants’ hit rates revealed the main effects of valence and direction. Participants responded correctly more often when positive words were displayed, and when the correct decision was to avoid valence and direction also interacted to predict accuracy. Participants made fewer errors approaching positive words than avoiding them (t(92) = 2.66, *p* = .008, *d* = 0.37), and fewer errors avoiding than approaching negative words (t(92) = 3.97, *p* < .001, *d* = 0.48). The difference between the magnitudes of the positive and negative conflict effects was negligible: t(89) = 1.27, *p* = .206, *d* = 0.13.

**TABLE 1:** Hit rates and reaction times by word valence (positive vs. negative) and response type (approach vs. avoid)

		Positive words		Negative words	
		Approach	Avoid	Approach	Avoid
Accuracy (%)	*M*	97.48%	94.53%	93.03%	97.45%
	*SD*	6.00%	12.09%	13.43%	5.99%
Reaction time (ms)	*M*	831.80	1055.95	1071.47	929.95
	*SD*	141.76	175.94	220.13	165.58

#### 3.1.2 Reaction times

We found main effects of valence and direction on reaction times, *F*(1,273) = 28.83, *p* < .001 and *F*(1,273) = 14.23, *p* < .001, respectively. These main effects were qualified by an interaction, *F*(1,273) = 339.35, *p* < .001. Participants approached positive words faster than they avoided them (t(89) = 16.64, *p* < .001, *d* = 1.75). Similarly, people avoided negative words faster than they approached them (t(89) = 8.17, *p* < .001, *d* = 0.86). The effect of positive conflict (avoid vs. approach positive words) was greater than the effect of negative conflict (approach vs. avoid negative words), t(89) = 4.82, *p* < .001, *d* = 0.50. To summarize, participants were slower in both conflicting conditions, but especially so when they avoided rather than approached positive words. Participants were not significantly faster at avoiding positive words than at approaching negative words (t(92) = 1.08, *p* = .27, *d* = 0.07). However, they approached positive words faster than they avoided negative words (t(92) = 6.88, *p* < .001, *d* = 0.62).

### 3.2 Event‐related potentials

The amplitude of the P1 and the N1 was insensitive to valence, direction, and their interaction. The amplitude of the N450 and CSP exhibited valence × direction interactions (see Table 2).

**TABLE 2:** Results of ANOVAs investigating the effects of word valence (positive, negative) and direction (approach, avoid) on the N450 and CSP, but not on the P1 or N1

Component	Effect	*F*	*p*	*p*Sidak	*ges*
P1	Valence	0.00	.98	1	0.00
	Direction	0.30	.59	1	0.00
	valence × direction	0.00	.98	1	0.00
N1	Valence	3.55	.06	.54	0.01
	Direction	2.17	.14	.85	0.01
	valence x direction	0.57	.45	1	0.00
N450	Valence	0.78	.38	1	0.00
	Direction	0.80	.37	1	0.00
	valence × direction	9.27	.00	.04	0.02
CSP	Valence	3.11	.08	.64	0.01
	Direction	11.59	.001	.01	0.07
	valence × direction	14.63	.0002	.003	0.04

*Note*. *ges* = Generalized Eta‐Squared measure of effect size (Bakeman, 2005); Numerator *df* = 1; Denominator *df* = 83.

**FIGURE 2:**
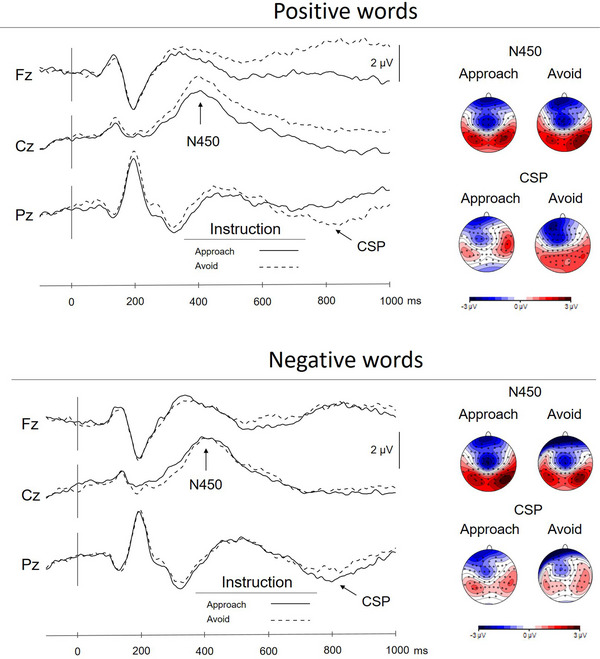
Sample ERPs from midline electrodes. Avoiding positive words elicited a larger central N450 and a more pronounced conflict slow potential than approaching positive words.

The N450 and the CSP were larger for the positive conflict conditions, (i.e., when participants approached positive words than when they avoided them; see Table 3 and Figure 2). To avoid negative and approach negative conditions did not differ according to established criteria of statistical significance. The mean amplitudes for each component and condition are reported in Suppporting Information Table S2.

**TABLE 3:** Each ERP component's sensitivity to movement direction as a function of stimulus valence

		*t*	*p*	*p*_Sidak_	*d*
Positive words: Avoid > Approach	P1	−0.80	.43	.99	−0.08
	N1	2.22	.03	.21	0.23
	N450	2.92	.004	.03	0.31
	CSP	5.00	.000002	.00002	0.53
Negative words: Approach > Avoid	P1	0.96	.34	.96	0.10
	N1	‐0.71	.48	.99	‐0.08
	N450	1.42	.16	.75	0.15
	CSP	‐0.31	.75	.99	−0.03

*Note*. Positive *t* and *d* values indicate higher amplitudes in the incongruent than in the congruent condition. Positive conflict (avoiding vs. approaching positive words) was associated with a larger N450 and CSP. We did not obtain the effect for negative conflict (approaching vs. avoiding negative words); CSP = conflict slow potential.

To summarize, the results supported our hypothesis that compared with negative conflict, positive conflict leads to stronger and more reliable effects, both behaviorally and neutrally. Positive conflict led to more pronounced reaction time slowing than negative conflict, and only positive conflict was associated with enhanced N450 and CSP amplitudes.

### 3.3 Additional analyses

Hit rates and reaction times to the negative words varied more than those to the positive words did. That higher variability may have jittered the signal, causing ERPs elicited by negative words to be systematically smaller. To address whether this was the case, we correlated participants’ ERP amplitude strengths with the standard deviations of their reaction times. We did this for positive and negative words separately. The results indicated that the amplitudes of the P1, the N1, the N450, and the CSP were unrelated to the variance in people's reaction times (see Supporting Information Figure S1).

Based on prior findings that anxious participants are more sensitive to conflict (Leue et al., 2012), we investigated the distribution of trait anxiety in our sample and determined whether it affected reaction times and electrophysiological responses to conflict. We did this to determine whether our findings could be an artifact of high trait anxiety in our sample. The sample's average score on the State‐trait anxiety inventory (STAI; Spielberger et al., 1970) was 40.39 (*SD* = 9.27), which is commonly classified as “moderate anxiety” (Kayikcioglu et al., 2017). The sample was right‐skewed, indicating that more participants were below than above that average. Trait anxiety was not able to predict the effect of conflict on the CSP. Participants with lower trait anxiety required more time to resolve positive conflict, did not require more or less time to resolve negative conflict (see Supporting Information Figure S2). These results indicate that our sample was not exceptionally anxious, and that the effects we found were caused or enhanced by high anxiety levels. If anything, low anxiety may have enhanced the effects of positive conflict.

## 4 DISCUSSION

Based on the proposition that positive valence is more consistently linked to approach than negative valence is linked to avoidance, we hypothesized that there should be stronger SRC effects with positive stimuli than with negative stimuli in an approach‐avoidance task. Using a manikin task, we indeed found that participants approached positive words faster than they avoided them. They also avoided negative words faster than they approached them, but the former effect was larger. ERPs also yielded stronger SRCs with positively valent words. Avoiding positive stimuli elicited a larger N450 and CSP. ERP differences between approaching and avoiding negative words were smaller and nonsignificant.

In our opinion, the main reason for these valence‐dependent effects of conflict is that positive valence is more closely linked to approach than negative valence is linked to avoidance. First, this is because not all negative emotions are avoidance‐related. Anger, for example, is approach‐related (Carver & Harmon‐Jones, 2009; Harmon‐Jones, 2004). Second, approaching and avoiding themselves also differ. For example, one can avoid actively by running away, or passively by doing nothing (Gray & McNaughton, 2000) but one can only approach actively. This echoes in psycholinguistic evidence that negative affective language is more diversified because people can experience many different negative affective yet fewer different positive affective states (Semin & Fiedler, 1991; Schrauf & Sanchez, 2004).

To our knowledge, this is the first study to explicitly compare the neurophysiological consequences of positive and negative approach‐avoidance conflict, and the first neurophysiological study of the manikin task. Because the positive and negative words used in the manikin task are not conflicting per se, conflict in this task is unlikely to arise at the stimulus level. Instead, it should arise at the response level, where there is competition between default approach or avoidance tendencies and the intention to override them. Tasks that feature response‐level conflict tend to evoke a positive slow wave, which is often referred to as the CSP in the second half‐second after stimulus presentation (e.g., Heidlmayr et al., 2020; Hanslmayr et al., 2008). The manikin task seems to be no exception, as it also yielded a CSP that clearly differentiated words that had to be approached and avoided. Somewhat unexpectedly, positive conflict also raised the amplitude of the N450, which is believed to reflect conflict at the stimulus level (Szücz & Soltéz, 2012). One way to reconcile these findings with existing ERP literature is to think of conflict in the manikin task as a case of interference suppression (Friedman & Miyake, 2004), whereby the default tendency to approach positive and avoid negative stimuli is a distractor that interferes with the (exactly opposite) task demands. Tasks that require interference suppression often evoke N450s as well as CSPs (Pires et al., 2014; for a review, see Heidlmayr et al., 2020).

The fact that in the present study, positive and negative conflict did not affect the earlier P1 and N1 components may be due to the way in which we manipulated stimulus‐response conflict. We presented conflicting and nonconflicting stimuli in separate blocks rather than intermixed. Hence, although participants had to implement control in the conflicting trials, there was no need to shift dynamically between controlled and uncontrolled modes of responding, and thus, a low demand for performance monitoring (MacDonald et al., 2000; Yeung et al., 2004). When using intermixed instead of blockwise presentation, one may, however, find early, monitoring‐related ERP components to participate in resolving conflict in the manikin task.

### 4.1 Limitations and outlook

Our results indicate stronger effects of positive than negative conflict in a word‐based approach‐avoidance task. One limitation of this study is that we used a relatively small set of positive and negative words that may not represent all existing positive and negative words. Using more representative stimulus sets may lead to different, perhaps even stronger results. Using more fine‐grained stimulus sets that differentiate different kinds of negative affect may also provide interesting opportunities to test our hypothesis. For example, although anger is a negative approach‐related affect, it would be interesting to see whether people find it easier to approach rather than avoid anger‐related words. Another limitation relates to the fact that regulation in the manikin task (and other approach‐avoidance tasks) is instructed rather than spontaneous. How much can we learn about the neural basis of regulation from instructed regulation? This question pertains not only to the present study, but to all studies requiring instructed overriding of impulsive response tendencies where the motive for overriding congruent response tendencies is imposed by the experimenter. People's motives for regulation are often internal. Researchers have begun to address similar differences between instructed and self‐directed emotion regulation (Mauss et al., 2007, 2006). Future studies are needed to explore commonalities and differences related to instructed versus self‐directed self‐regulation as well as different modes of self‐regulation (Kuhl, 2000).

### OPEN PRACTICE STATEMENT

Behavioral data, EEG data and study materials, as well as the code used for data analysis are available at https://osf.io/73pmw.

### PEER REVIEW

The peer review history for this article is available at https://publons.com/publon/10.1002/brb3.3008

## Supporting information


**Supplemental Table 1** A complete list of the positive and negative stimulus words (and their English translations) used in the experiment

**Supplemental Table 2**: *Mean ERP amplitudes as a function of stimulus valence (positive vs. negative) and response (avoid vs. approach)*

**Supplemental Figure 1**: Variability (SD) in participants’ response times to positive words (top row) and negative words (bottom row) was not significantly correlated with the amplitude of the P1 (first column), N1 (second column), N450 (third column) and conflict slow potential (CSP; fourth column)

**Supplemental Figure 2**: I) The distribution of participants’ trait anxiety scores, as measured by Form Y of the State‐trait anxiety inventory (STAI; Spielberger et al., 1970). II‐III) There were no significant correlations between trait anxiety and the amplitude of the Conflict slow potential (CSP). IV) Compared to highly anxious participants, participants with low trait anxiety required more time to avoid positive words than to approach them. V) Trait anxiety did not affect how much time participants took to approach than avoid negative words.

## Data Availability

The data that support the findings of this study are available from the corresponding author upon reasonable request.

## Footnotes

1 In principle, conflict in approach‐avoidance tasks may also stem from ambiguous words whose valence depends on the context. For example, the word “power” may be positive or negative, depending on whether one approves of whether and how that entity exerts that power. However, designers of approach‐avoidance tasks are aware of that problem, and typically select stimulus words that are clearly positive and negative.
