# Supplementary material for: Conflict in a word‐based approach‐avoidance task is stronger with positive words
Source: Brain Behav. 2023 May 11;13(6):e3008. doi: 10.1002/brb3.3008 (PMC10275559; doi:10.1002/brb3.3008)

**Supplemental Figure 1**

*Variability (SD) in participants’ response times to positive words (top row) and negative words (bottom row) was not significantly correlated with the amplitude of the P1 (first column), N1 (second column), N450 (third column) and conflict slow potential (CSP; fourth column)*


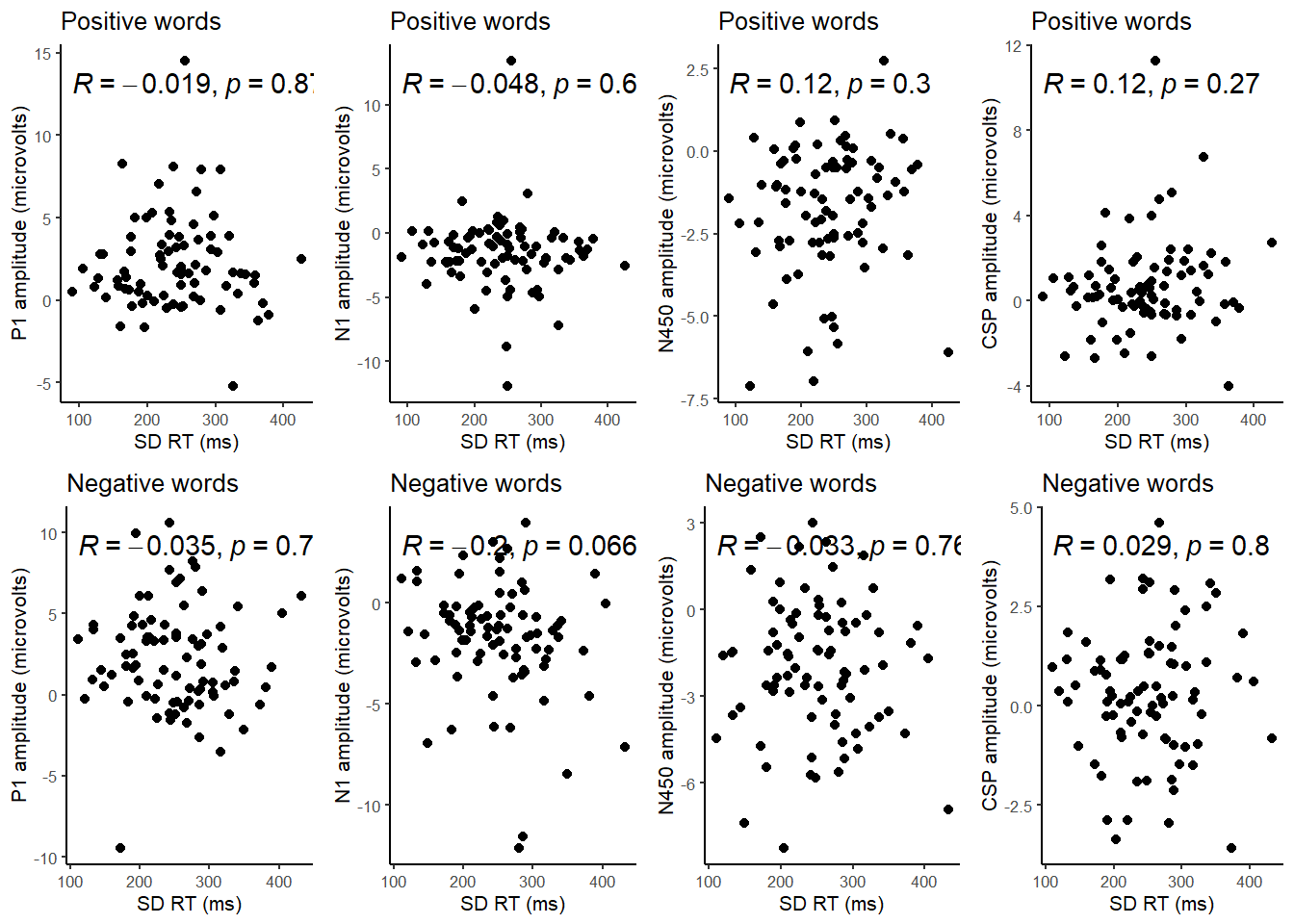

Supplement: Supplementary file 1 — Supplemental Table 1 A complete list of the positive and negative stimulus words (and their English translations) used in the experiment [file BRB3-13-e3008-s003.docx]
