# Supplementary material for: Conflict in a word‐based approach‐avoidance task is stronger with positive words
Source: Brain Behav. 2023 May 11;13(6):e3008. doi: 10.1002/brb3.3008 (PMC10275559; doi:10.1002/brb3.3008)
Supplement: Supplementary file 4 — Supplemental Figure 2: I) The distribution of participants’ trait anxiety scores, as measured by Form Y of the State‐trait anxiety inventory (STAI; Spielberger et al., 1970). II‐III) There were no significant correlations between trait anxiety and the amplitude of the Conflict slow potential (CSP). IV) Compared to highly anxious participants, participants with low trait anxiety required more time to avoid positive words than to approach them. V) Trait anxiety did not affect how much time participants took to approach than avoid negative words. [file BRB3-13-e3008-s002.docx]

| **Supplemental Table 2**  *Mean ERP amplitudes as a function of stimulus valence (positive vs. negative) and response (avoid vs. approach)* | | | | | |
| --- | --- | --- | --- | --- | --- |
|  |  | *M* | *SD* | CIl | CIu |
| Avoid positive | P1 | 1.13 | 2.02 | 0.74 | 1.55 |
|  | N1 | -2.43 | 1.94 | -2.83 | -2.05 |
|  | N450 | -1.96 | 1.73 | -2.35 | -1.62 |
|  | CSP | 0.19 | 2.65 | -0.35 | 0.69 |
| Approach positive | P1 | 1.29 | 2.21 | 0.83 | 1.73 |
|  | N1 | -2.00 | 2.02 | -2.48 | -1.59 |
|  | N450 | -1.48 | 1.70 | -1.82 | -1.13 |
|  | CSP | -1.34 | 2.82 | -1.92 | -0.77 |
| Approach negative | P1 | 1.30 | 1.92 | 0.92 | 1.70 |
|  | N1 | -1.86 | 1.75 | -2.22 | -1.51 |
|  | N450 | -1.75 | 1.52 | -2.05 | -1.45 |
|  | CSP | -0.94 | 2.47 | -1.44 | -0.41 |
| Avoid negative | P1 | 1.08 | 2.26 | 0.63 | 1.52 |
|  | N1 | -1.97 | 1.81 | -2.38 | -1.60 |
|  | N450 | -1.56 | 1.46 | -1.87 | -1.28 |
|  | CSP | -0.85 | 2.98 | -1.48 | -0.26 |
| Notes: CIl = lower limit of the 95% confidence interval; CIu = upper limit of the 95% confidence interval; CSP = conflict slow potential. | | | | | |
